# Author Correction: Fine mapping spatiotemporal mechanisms of genetic variants underlying cardiac traits and disease

**DOI:** 10.1038/s41467-023-40048-9

**Published:** 2023-07-20

**Authors:** Matteo D’Antonio, Jennifer P. Nguyen, Timothy D. Arthur, iPSCORE Consortium, Angelo D. Arias, Timothy D. Arthur, Paola Benaglio, W. Travis Berggren, Victor Borja, Juan Carlos Izpisua Belmonte, Megan Cook, Matteo D’Antonio, Christopher DeBoever, Kenneth E. Diffenderfer, Margaret K. R. Donovan, KathyJean Farnam, Kelly A. Frazer, Kyohei Fujita, Melvin Garcia, Olivier Harismendy, Benjamin A. Henson, David Jakubosky, Kristen Jepsen, He Li, Hiroko Matsui, Naoki Nariai, Jennifer P. Nguyen, Daniel T. O’Connor, Jonathan Okubo, Athanasia D. Panopoulos, Fengwen Rao, Joaquin Reyna, Bianca Salgado, Erin N. Smith, Josh Sohmer, Shawn Yost, William W. Young Greenwald, Hiroko Matsui, Agnieszka D’Antonio-Chronowska, Kelly A. Frazer

**Affiliations:** 1 Department of Pediatrics, University of California San Diego, La Jolla, CA 92093 USA; 2 Division of Biomedical Informatics, University of California, San Diego, La Jolla, CA 92093 USA; 3 Institute of Genomic Medicine, University of California San Diego, 9500 Gilman Dr, La Jolla, CA 92093 USA; 4 Bioinformatics and Systems Biology Graduate Program, University of California, San Diego, La Jolla, CA 92093 USA; 5 Biomedical Sciences Graduate Program, University of California, San Diego, La Jolla, CA 92093 USA; 6 Stem Cell Core, Salk Institute for Biological Studies, La Jolla, CA 92037 USA; 7 Gene Expression Laboratory, Salk Institute for Biological Studies, La Jolla, CA 92037 USA; 8 Department of Medicine, University of California, San Diego, La Jolla, CA 92093 USA

**Keywords:** Gene expression, Quantitative trait, Personalized medicine

Correction to: *Nature Communications* 10.1038/s41467-023-36638-2, published online 28 February 2023

The original version of this article included an incomplete list of the iPSCORE consortium author members. The incorrect version read “Matteo D’Antonio, Jennifer P. Nguyen, Timothy D. Arthur, Hiroko Matsui, Agnieszka D’Antonio-Chronowska & Kelly A. Frazer”. The correct version replaces this list with “Angelo D. Arias, Timothy D. Arthur, Paola Benaglio, W. Travis Berggren, Victor Borja, Juan Carlos Izpisua Belmonte, Megan Cook, Matteo D’Antonio, Agnieszka D’Antonio-Chronowska, Christopher DeBoever, Kenneth E. Diffenderfer, Margaret K.R. Donovan, KathyJean Farnam, Kelly A. Frazer, Kyohei Fujita, Melvin Garcia, Olivier Harismendy, Benjamin A. Henson, David Jakubosky, Kristen Jepsen, He Li, Hiroko Matsui, Naoki Nariai, Jennifer P. Nguyen, Daniel T. O’Connor, Jonathan Okubo, Athanasia D. Panopoulos, Fengwen Rao, Joaquin Reyna, Bianca Salgado, Erin N. Smith, Josh Sohmer, Shawn Yost, William W. Young Greenwald”. In addition, three affiliations were added to the author affiliation list: 6. Stem Cell Core, Salk Institute for Biological Studies, La Jolla, CA 92037, USA (W. Travis Berggren, Kenneth E. Diffenderfer), 7. Gene Expression Laboratory, Salk Institute for Biological Studies, La Jolla, CA 92037, USA (Athanasia D. Panopoulos, Juan Carlos Izpisua Belmonte) and 8. Department of Medicine, University of California, San Diego, La Jolla, CA 92093, USA (Daniel T. O’Connor and Fengwen Rao). In addition, the author contribution section was updated to include the iPSCORE consortium, replacing the previous version “K.A.F., A.D.C., and M.D. conceived the study. M.D., J.P.N., T.D.A., and H.M. performed the analyses. H.M. performed quality check on RNA-seq samples. K.A.F. and A.D.C. oversaw the study. M.D. and K.A.F. prepared the manuscript” with complete version “KAF, ADC, MD and iPSCORE consortium members conceived the study. MD, JPN, TDA, HM and iPSCORE consortium members performed the analyses. HM performed quality check on RNA-seq samples. KAF, ADC and iPSCORE consortium members oversaw the study. MD, JPN and KAF prepared the manuscript.” This has been corrected in both the PDF and HTML versions of the Article.

