# Supplementary material for: Fine mapping spatiotemporal mechanisms of genetic variants underlying cardiac traits and disease
Source: Nat Commun. 2023 Feb 28;14:1132. doi: 10.1038/s41467-023-36638-2 (PMC9975214; doi:10.1038/s41467-023-36638-2)
Supplement: Supplementary file 1 — Supplementary Information [file 41467_2023_36638_MOESM1_ESM.pdf]

Supplementary Notes

Supplementary Note 1: Enrichment analysis of gene/antisense pairs sharing the same eQTL signal

In total, we tested 163 pairs of genes and their associated antisense RNAs and observed that 47 pairs shared the same eQTL signal. We examined the enrichment of genes that share an eQTL signal with their antisense RNA compared to those that do not. We found that pairs that share an eQTL were enriched for splice acceptor sites and promoter regions while those that have distinct signals were enriched intronic and splice donor regions (Supplementary Fig. 6A). This indicates that as previously observed [1,2], there is a coordinated regulatory mechanism of promoters shared by eGenes-antisense pairs of genes.

Of the 47 pairs that shared the same eQTL signal, we found 12 whose eQTL acted in opposite directions on the expression of the eGene and its antisense RNA (Supplementary Fig. 7, Supplementary Fig. 8). We next tested whether the eQTLs for correlated and anticorrelated eGene/antisense pairs were associated with different types of regulatory elements and found that correlated pairs were enriched for intergenic regions while anti-correlated pairs were enriched for splice acceptor and donor sites and exonic regions (Supplementary Fig. 6B). The 12 pairs with opposite effects included two genes whose antisense RNA is known to be involved in the negative regulation of gene expression: *TRAPPC12/TRAPPC12-AS1* and *ABCF2/ABCF2-AS1* [3,4]. These results suggest that, for these 12 pairs, the eQTLs may increase the regulatory effect of the antisense RNA, whose change in expression (increase/decrease) results in the opposite expression change (decrease/increase) on their target gene.

Supplementary Note 2: Validation of enrichment of tissue-specific eQTLs in the cardiac GWAS signals

Different approaches for identifying tissue-associated eQTLs

Multiple studies have shown that eQTLs can be shared across multiple tissues [5-9], but there is no consensus on a specific approach to identify shared eQTLs versus eQTLs that are specific or associated with a specific tissue. The simplest approach, introduced by GTEx [6], includes performing eQTL analyses independently in each tissue and then determining whether an eQTL is observed in one or multiple tissues. More advanced methods, such as multivariate adaptive shrinkage (mash) [7], compare effect sizes of the same variants across multiple tissues. The major advantage of mash is improving the power of eQTL detection in all tissues by combining the effect sizes of the same variant across tissues [8]. As input, mash requires effect sizes (β) and their standard errors of genes obtained by conducting condition-by-condition analyses. Only genes with available effect sizes for all conditions are considered. Combined method approaches, such as the one used in this study and FastGxC [9], distinguish between tissue-specific and tissue-shared eQTLs using linear mixed models. These methods are performed in two steps: in the first-step a combined eQTL analysis is conducted using all tissues and then in the second-

step context-specific eQTLs are identified using an interaction test between genotype and each of the contexts. Of note, while mash's advantage is improved power of detecting eQTLs, it can also be used to identify tissue-specific eQTLs [8]; on the contrary, the second-step in the combined eQTL methods is specifically aimed at identifying tissue-specific eQTLs. Additionally, mash can only work for categorical features (such as developmental stage, organ or tissue: each sample can be described as belonging to only one of each developmental stage, organ or tissue), but not continuous (cell type proportions) features; whereas the combined analysis approach can work for both categorical and continuous features.

Performing mash

Mash requires effect sizes and their standard errors in each tissue as input. To have a direct comparison between mash and our outputs for validation, we initially performed an eQTL analysis on each tissue separately using limix and the same covariates as our combined eQTL approach (see Supplementary Data 9 and Methods section "Validation of tissue-associated eQTLs"). We then selected from our combined eQTL analysis the lead variant of each of the 18,030 eQTLs (11,692 primary and 6,338 conditional, Figure 1A), obtained their effect sizes and standard errors for each tissue from the single-tissue eQTL analyses, and used these values to perform mash with default parameters. We annotated each eQTL signal as active in a tissue if the local false sign rate (LFSR) < 0.05 (Supplementary Data 10).

Comparing eQTLs obtained with different methods

Using the single-tissue eQTL analyses we found varying numbers of eGenes and eQTLs across tissues: 1) aorta: 9,812 eGenes, and 13,029 eQTL signals (3,217 conditional eQTLs); atrium: 7,148 eGenes, and 8,770 eQTL signals (1,622 conditional eQTLs); ventricle: 5,951 eGenes, and 7,353 eQTL signals (1,402 conditional eQTLs); coronary artery: 5,458 eGenes, and 6,143 eQTL signals (685 conditional eQTLs); and iPSC-CVPC: 3,393 eGenes, and 3,682 eQTL signals (289 conditional eQTLs, Supplementary Data 9). The observed differences in the number of eQTLs in each tissue are likely due to power differences across the tissues from sample numbers and tissue heterogeneity.

We next examined the mash results obtained using the eQTLs (for eGenes, i.e., not for eIsoforms) from the first-step of our combined approach coupled with their effect sizes and standard errors for each tissue from the single-tissue eQTL analyses as input (Supplementary Data 10). Mash improved the detection of eQTLs active in each tissue compared with the single-tissue eQTL analysis for all tissues but aorta (1.45X times for atrium, 1.63X for ventricle, 1.89X for coronary artery and 2.40X for iPSC-CVPCs, Supplementary Fig. 13). Interestingly, the least-powered tissues (i.e. with the smallest number of unrelated samples: iPSC-CVPCs and coronary artery) had the largest increase in the number of eQTL signals detected, whereas the tissue with the largest number of tested samples (aorta) had a comparable number of eQTLs detected with the two methods. These results demonstrate that performing one combined eQTL analysis using all tissue samples and then identifying tissue associations results in increased power, compared with performing single-tissue eQTL analyses, as was previously suggested [5,7,8].

Of note, neither the traditional tissue-by-tissue eQTL analyses, nor the mash approach is able to detect cell type-associations, which can only be identified by using our interaction approach (described in the Methods section "Spatiotemporal eQTL

mapping”). Therefore, while mash is useful to determine the tissue context of each eQTL, it cannot fully substitute for our combined analysis approach, as it cannot be used to test cell type associations.

Using mash results to validate enrichment of tissue-specific eQTLs and GWAS signals

We sought to validate enrichments of the tissue-specific eQTLs and GWAS signals shown in Figure 5E-G and Supplementary Fig. 9. Using the eQTLs detected by mash for each tissue, we performed enrichment tests across the five cardiac GWAS traits. We used varying LFSR thresholds to define whether a gene was expressed in a given tissue, and observed similar enrichments to those obtained using our combined analysis approach (Supplementary Fig. 14):

- Myocardial infarction does not have strong associations with any tissue;
- QRS duration does not have strong associations with any tissue;
- Atrial fibrillation is enriched for eQTLs active in atrium and, at stringent LFSR thresholds, with ventricle (LFSR $\leq 1 \times 10^{-5}$) and with iPSC-CVPC (LFSR $\leq 1 \times 10^{-10}$);
- Pulse rate is enriched for eQTLs active in atrium and ventricle at all LFSR thresholds, and with aorta at most thresholds;
- Pulse pressure is enriched for eQTLs active in aorta at all thresholds, iPSC-CVPC at stringent LFSR thresholds (LFSR $\leq 1 \times 10^{-10}$), and coronary artery at loose LFSR thresholds (LFSR ≥ 0.05).

Supplementary Figures

Supplementary Fig. 1: Examples of associations between eQTL signals and cardiac stage

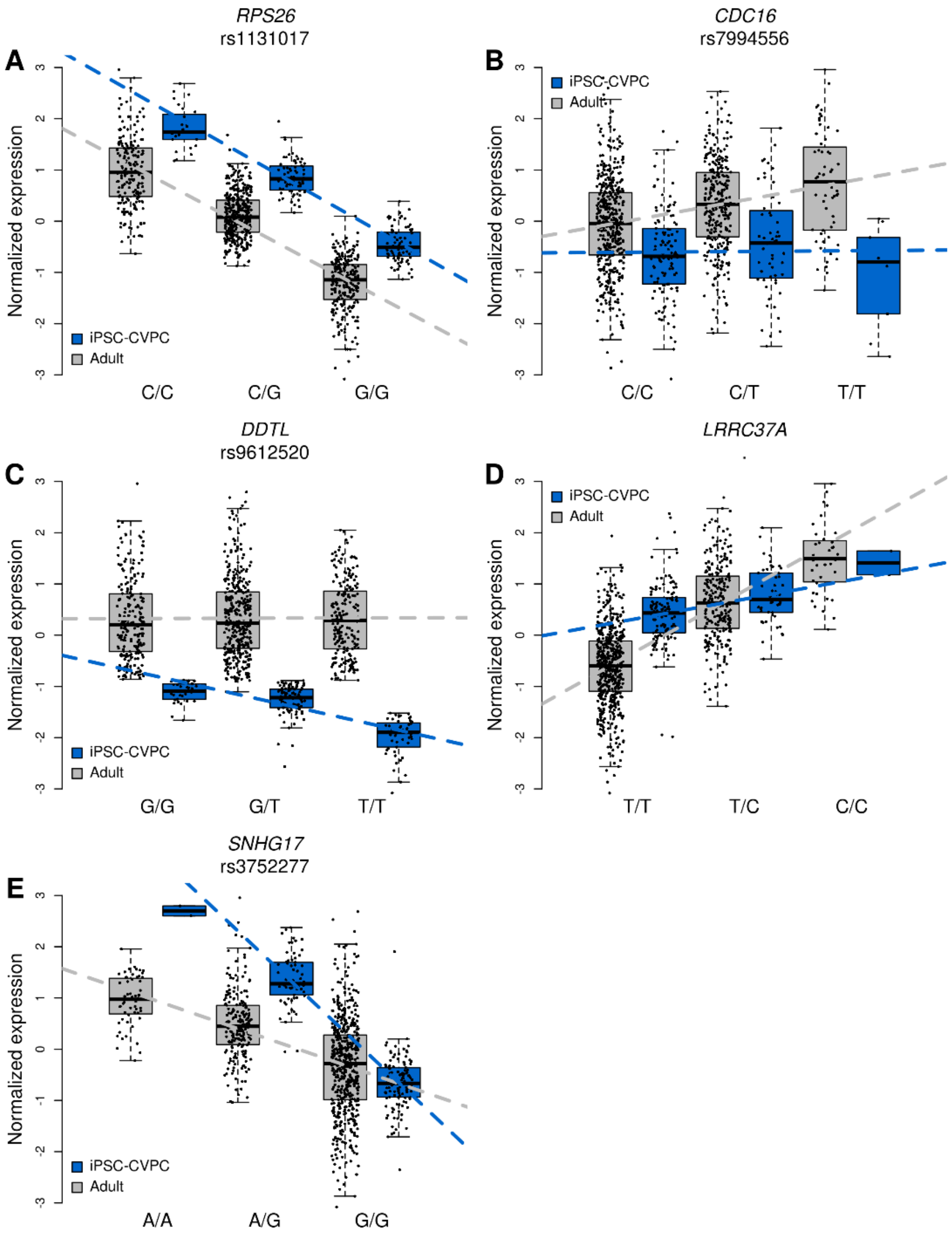

Examples of five association types between eQTLs and cardiac stage. For each eGene, boxplots (n = 966 samples) describe the normalized expression in iPSC-CVPCs (blue) and all other samples (i.e., adult cardiac samples; gray), grouped by genotype. The panels show examples of: (A) an eGene whose eQTL is shared across both cardiac stages; (B) an eGene whose eQTL is adult-specific: the association between genotype and gene expression is present in the adult cardiac samples but not in iPSC-CVPCs; (C) an iPSC-CVPC-specific eQTL: the association between genotype and gene expression is only present in iPSC-CVPCs; (D) an adult-associated eQTL: although an association between genotype and gene expression is present also in iPSC-CVPCs, it is significantly stronger in the adult samples; and (E) iPSC-CVPC-associated eQTL: while the genotype is associated with gene expression in both sets of samples, the eQTL is significantly stronger in iPSC-CVPCs.

The boxplots were built as follows: upper and lower edges represent the 25th and 75th percentiles and the middle line the median, vertical bars represent the distance from the 25th (or 75th) percentile minus (or plus) 1.5 times the interquartile range.

Supplementary Fig. 2: Examples of associations between eQTL signals and cardiac tissue (adult left ventricle)

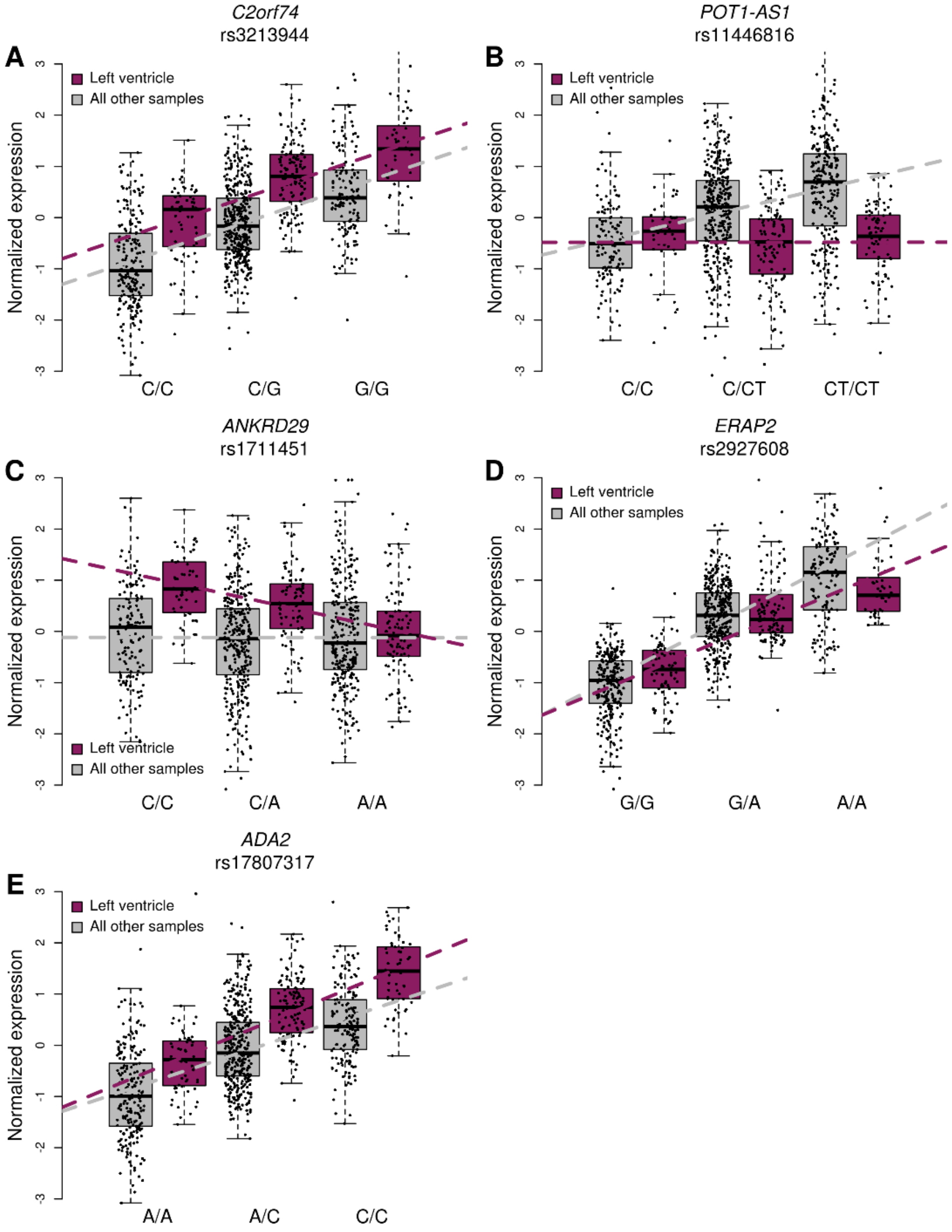

The Supplementary Fig. hows examples of five association types between eQTLs and left ventricle. For each eGene, boxplots (n = 966 samples) describe the normalized expression in adult left ventricle (maroon) and all other samples (i.e. all cardiac samples that are not left ventricle; gray), grouped by genotype. The panels show examples of: (A) an eGene whose eQTL is shared across cardiac tissues; (B) an eGene whose eQTL is specific to other cardiac tissues as there is no association between the genotype and gene expression in left ventricle; (C) a left ventricle-specific eQTL: the association between genotype and gene expression is present in left ventricle, but not in the other cardiac tissues; (D) an eQTL that is associated with other cardiac tissues: although an association between genotype and gene expression is present also in left ventricle, it is significantly stronger in the other samples; and (E) left ventricle-associated eQTL: while the genotype is

associated with gene expression in both left ventricle and other cardiac tissues, the eQTL is significantly stronger in left ventricle. The boxplots were built as follows: upper and lower edges represent the 25th and 75th percentiles and the middle line the median, vertical bars represent the distance from the 25th (or 75th) percentile minus (or plus) 1.5 times the interquartile range.

Supplementary Fig. 3: Associations between eQTL signals and cell type (cardiac muscle proportion)

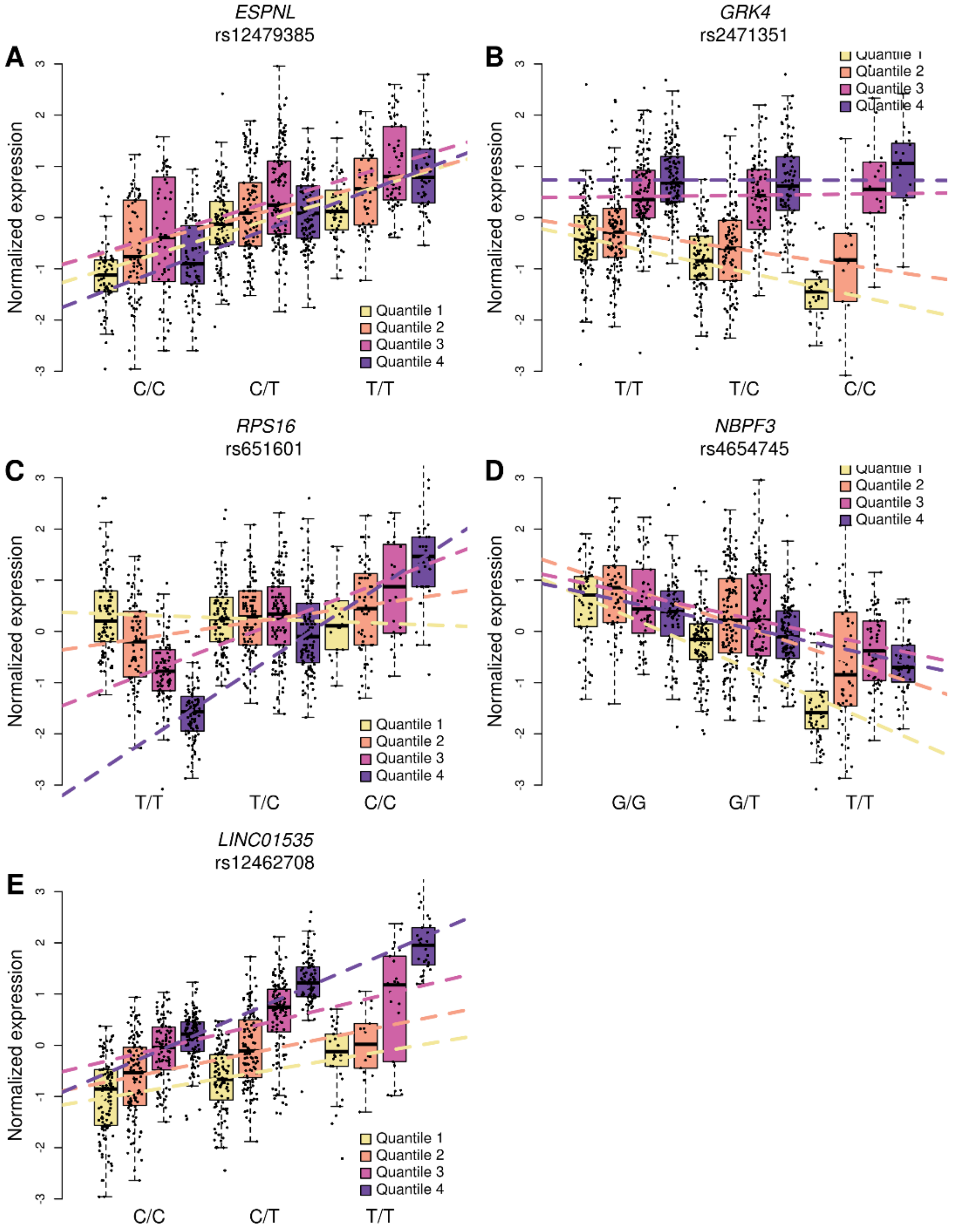

Examples of five association types between eQTLs and cell types. For each eGene, boxplots (n = 966 samples) describe the normalized expression divided into four quartiles according to their cardiac muscle proportion (yellow = low; purple = high), grouped by genotype. The panels show examples of: (A) an eGene whose eQTL is shared across cell types; (B) an eGene whose eQTL is specific to other cell types but is not associated with cardiac muscle: only samples in the bottom quartiles show an association between the genotype and gene expression; (C) a cardiac muscle-specific eQTL: the association between genotype and gene expression is only present in the top quartiles; (D) an eGene whose eQTL is associated with other cell types: while the association between genotype and gene expression is present in all quartiles, it is significantly stronger in the bottom quartiles; and (E) cardiac muscle-associated eQTL: while the genotype is associated

with gene expression in all quartiles, the eQTL is significantly stronger in the top quartiles. The boxplots were built as follows: upper and lower edges represent the 25th and 75th percentiles and the middle line the median, vertical bars represent the distance from the 25th (or 75th) percentile minus (or plus) 1.5 times the interquartile range.

Supplementary Fig. 4: Enrichment of cell type- eQTLs for corresponding cell type- snATAC peaks

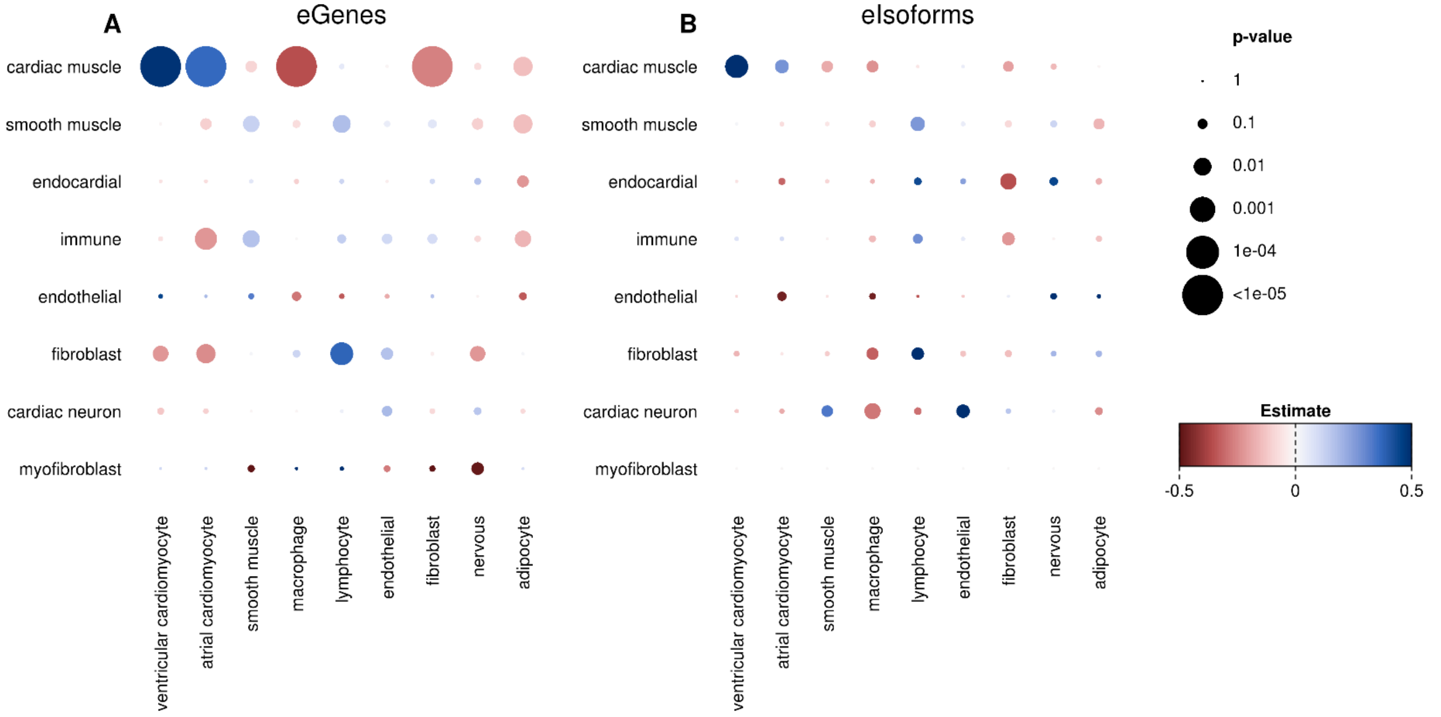

Bubble plots of enrichments of eQTLs for (A) eGenes and (B) eIsoforms associated with each cell type (rows) and the relative accessibility score of nine cardiac cell types obtained from adult cardiac snATAC-seq peaks from an independent study [10]. Estimates and p-values were calculated using the *t.test* (paired, two-sided) function in R.

Supplementary Fig. 5: Correlation between GTEx myocyte iQTLs and context-associated eQTLs

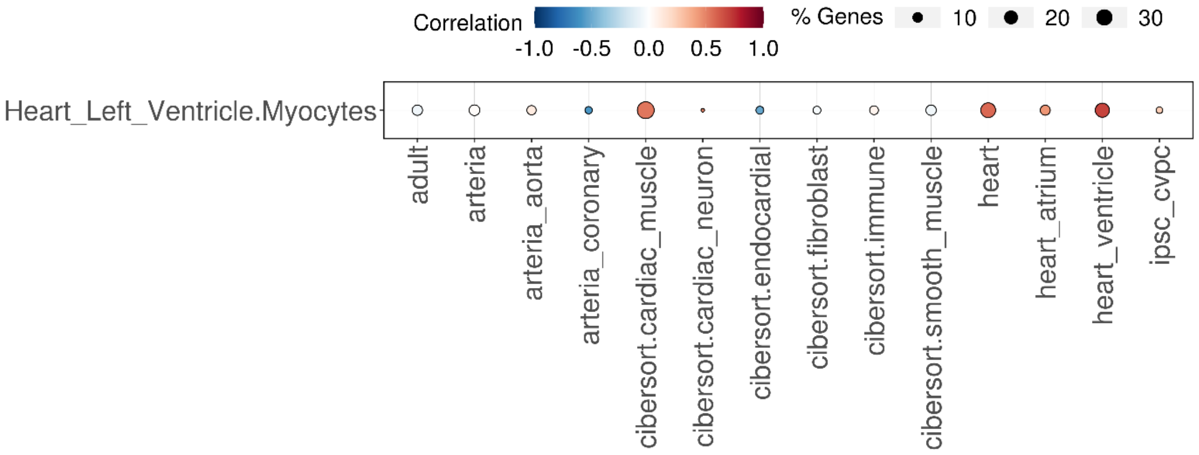

This Supplementary Fig. hows the correlation of effect sizes between interaction eQTLs (iQTLs) computed by GTEx for myocytes in the heart left ventricle [11] and each of the context-associated eQTLs identified in this study. We observed a positive association between myocyte iQTLs and eQTLs associated with cardiac muscle, heart tissue, and heart left ventricle. Color indicates strength and direction of the correlation. Point size indicates the percentage of overlapping eGenes with the GTEx iQTL dataset.

Supplementary Fig. 6: eGene/antisense pairs: Enrichment for different regulatory elements

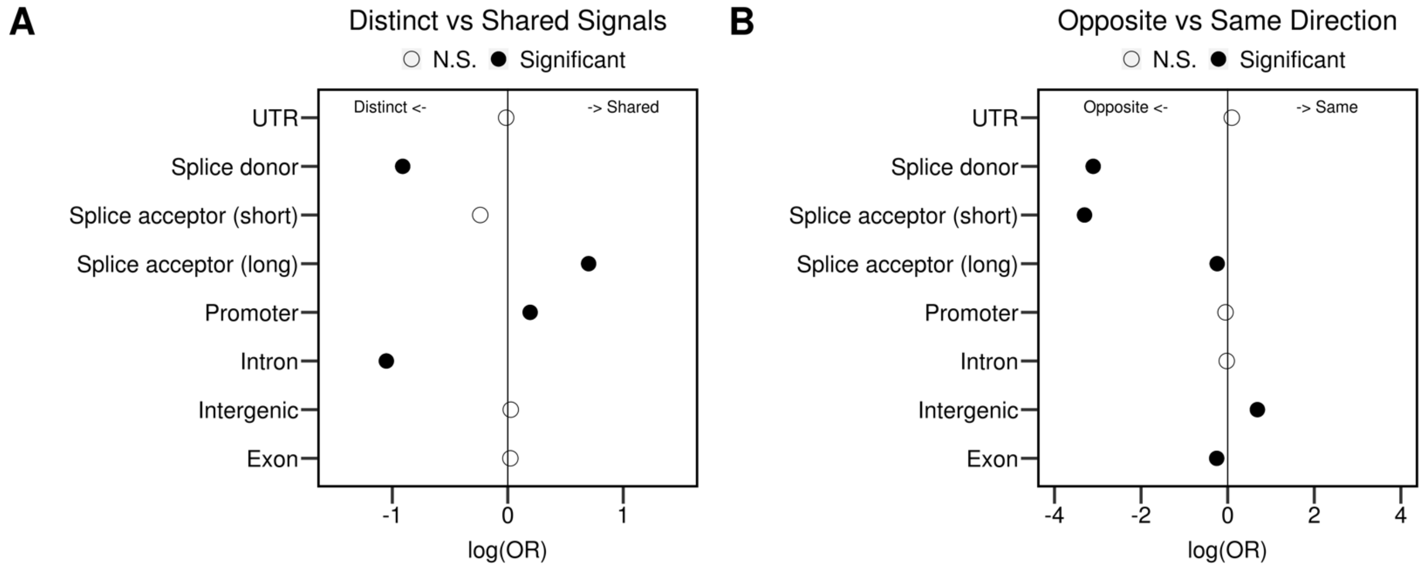

This Supplementary Fig. hows the enrichment of different regulatory elements between eGene/antisense pairs that shared the same eQTL signals (PP-H4 > 0.8) and those that had distinct signals (PP-H3 > 0.8) (Panel A) and between correlated and anti-correlated eGene/antisense pairs (Panel B). Enrichment was tested using a two-sided Fisher's Exact Test comparing the proportion of SNPs located in the genomic regions (see Methods). P-values were adjusted using Benjamini-Hochberg's method, and enrichments with q-values < 0.05 (closed points) were considered significant. X-axis represents the $\log_2$(odds-ratio). In Panel A, we compared 47 pairs that shared the same eQTL signals against 67 pairs that had distinct eQTL signals. In Panel B, we compared the 47 pairs that shared the same eQTL signals: 35 pairs with correlated directions against 12 pairs with anti-correlated directions.

Supplementary Fig. 7: Correspondence between the effect sizes of eGenes and their associated antisense RNA

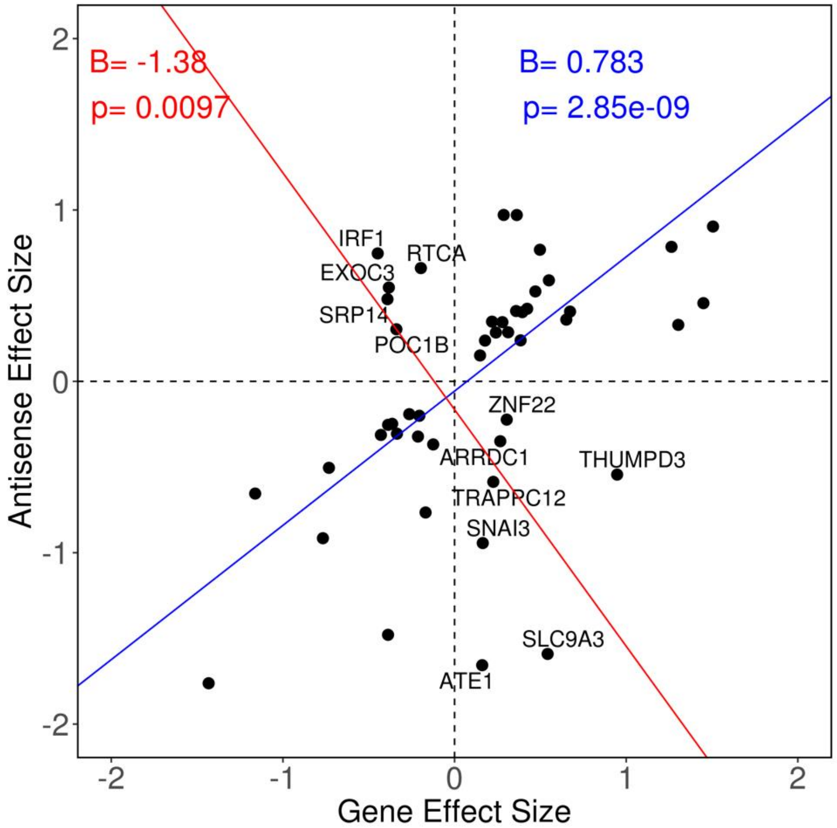

Scatterplot showing the eQTL effect sizes of eGenes (X axis) and their colocalizing antisense RNA (Y axis) for the 47 pairs that shared the same eQTL signals: 35 pairs with correlated directions and 12 pairs with anti-correlated directions

Supplementary Fig. 8: Effect sizes of anti-correlated eGenes and their antisense

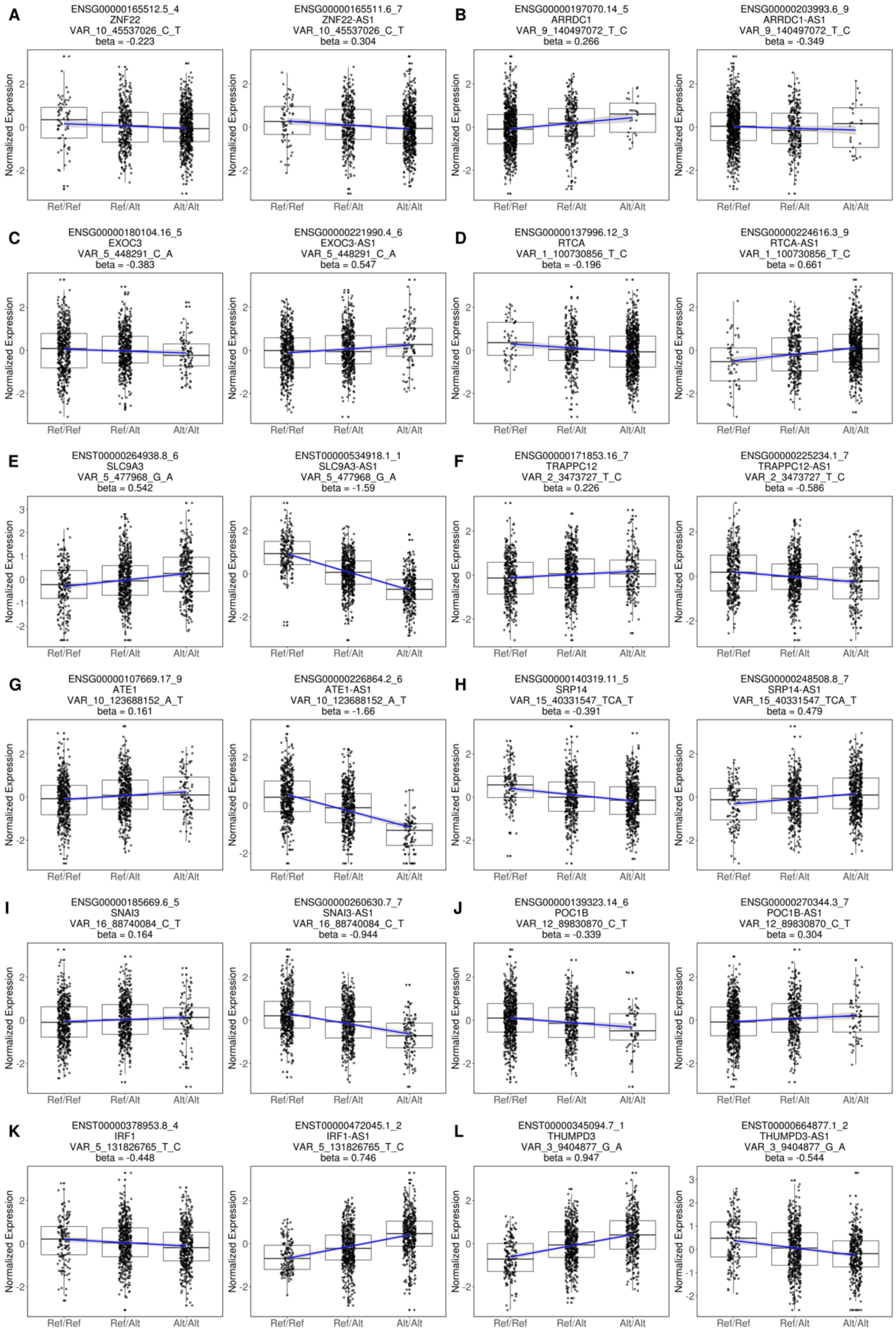

(A-L) Boxplots (n = 966 samples) showing the effect of the eQTL signals for the 12 pairs of associated eGenes/antisense RNA with anti-correlated directions. Each panel shows the eQTL signal for the eGene (left) and its antisense (right). For each plot, the Y axis describe the normalized expression grouped by genotype. The boxplots were built as follows: upper

and lower edges represent the 25th and 75th percentiles and the middle line the median, vertical bars represent the distance from the 25th (or 75th) percentile minus (or plus) 1.5 times the interquartile range.

Supplementary Fig. 9: Enrichment of GWAS traits for stage, organ and tissue associations

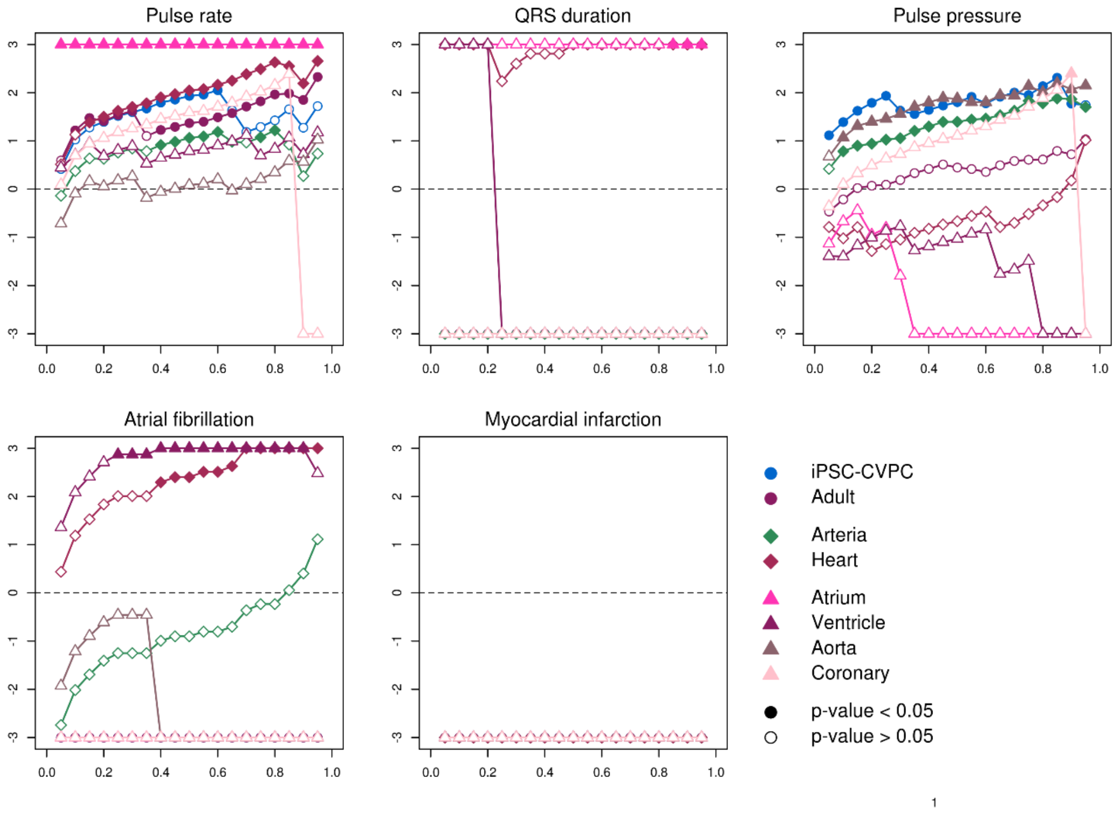

Here we show enrichment of GWAS traits for the spatiotemporal eQTLs obtained in this study. Enrichment for the associations between each of the five cardiac traits and diseases and stage, organ and tissue eQTL associations at multiple PP-H4 thresholds, as described in Donovan et al. [12]. Briefly, at each 0.05 PP-H4 increment between 0.05 and 0.95 (X axis), we tested the enrichment for each stage, organ or tissue for all the eGenes that had PP-H4 above the threshold using Fisher's exact test. The Y axis represents the $\log_2$ of the estimate, as calculated using the *fisher.test* function in R. Filled points represent p-values < 0.05. Log_2 estimate values > 3 or < -3 were set to +3 or -3, respectively. None of the stage, organ or tissue eQTLs colocalize with myocardial infarction signals, therefore all enrichment values are shown on the -3 line.

Supplementary Fig. 10: Enrichment of GWAS traits for cell type associations

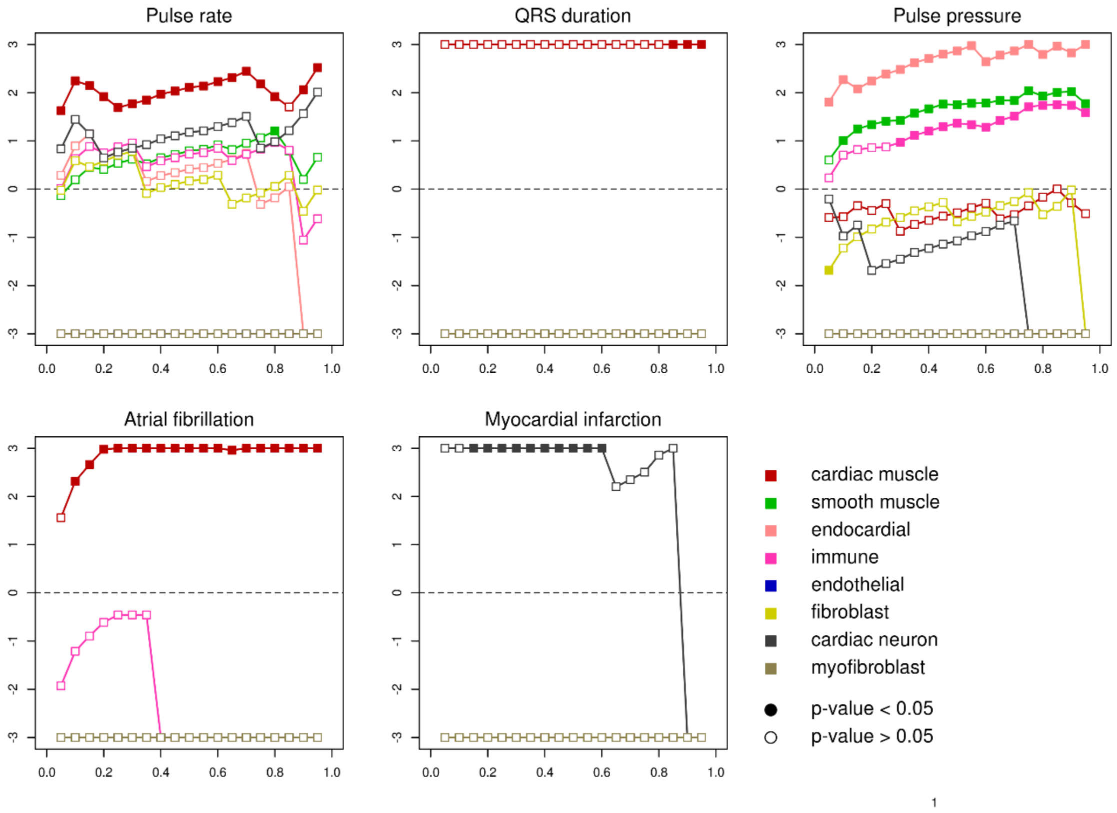

Here we show enrichment of GWAS traits for the spatiotemporal eQTLs obtained in this study. Enrichment for the associations between each of the five cardiac traits and diseases and cell type eQTL associations at multiple PP-H4 thresholds, as described in Donovan et al. [12], as described in Supplementary Fig. 9.

Supplementary Fig. 11: Colocalization between iPSC-CVPC- eQTLs and pulse pressure GWAS signals

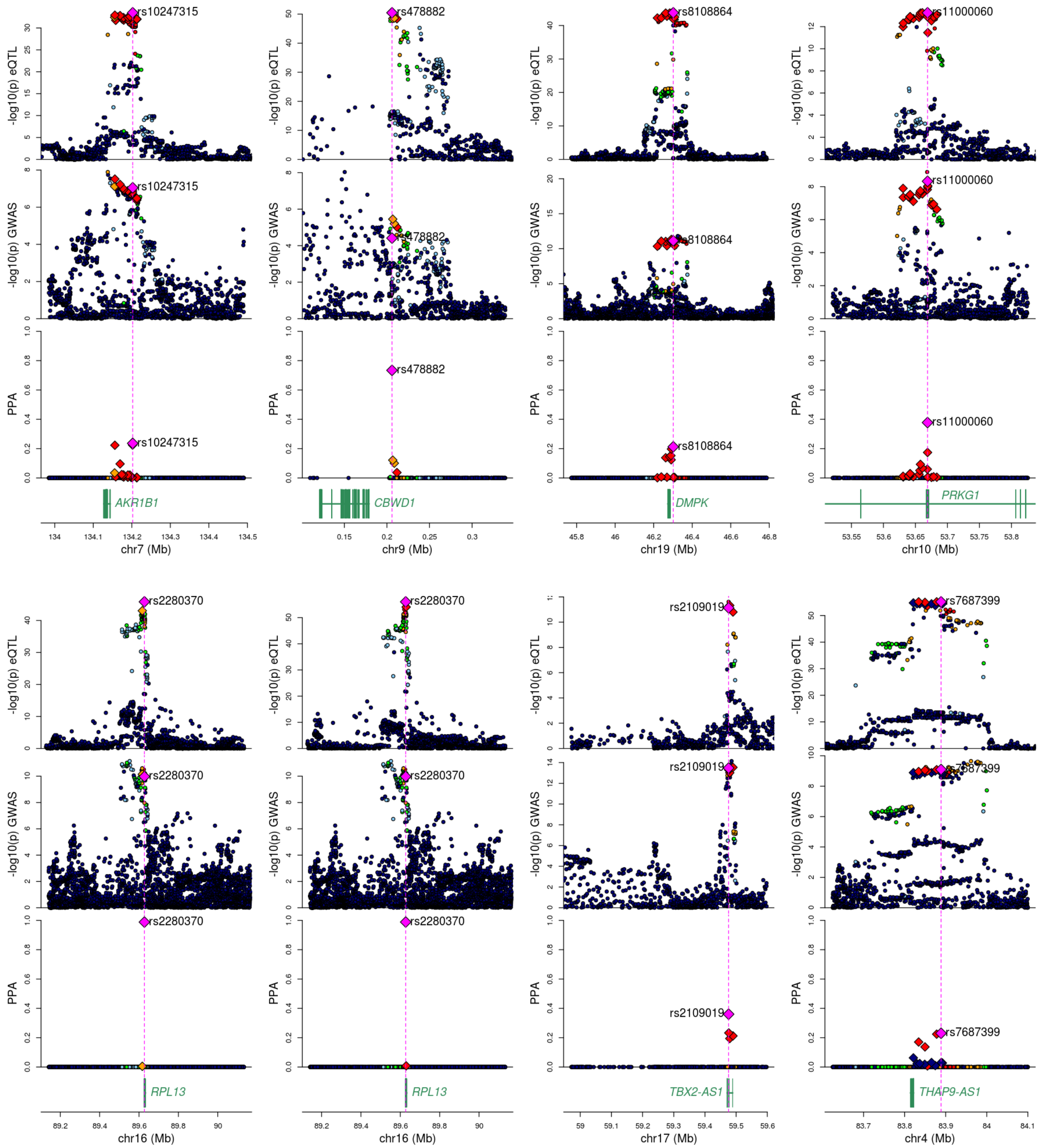

Plots showing -log$_{10}$(p-value) iPSC-CVPC- eQTL signals (top row), the colocalizing GWAS signals (pulse pressure, middle row) and the PPA of each variant (bottom row) for five eGenes and three eIsoforms. The iPSC-CVPC-eQTL signal with

lead variant rs2280370 is shared between the *RPL13* eGene and one of the *RPL13* eIsoforms (ENST00000565571.5_1). The other two iPSC-CVPC-eQTL signals associated with eIsoforms encode *DMPK* (ENST00000291270.9_3) and *PRKG1* (ENST00000643582.1_1). The lead variant (i.e. the variant with highest PPA of being causal for both the eQTL and GWAS signals) is shown as a magenta diamond. All non-lead variants included in the 99% credible set are shown as red diamonds. The other eVariants are color-coded based on their LD (R^2, calculated using the 1000 Genomes) with the lead eVariant: 1) red: $R^2 \geq 0.8$; 2) orange: $0.6 \leq R^2 < 0.8$; 3) green: $0.4 \leq R^2 < 0.6$; 2) cyan: $0.2 \leq R^2 < 0.4$; 2) dark blue: $R^2 < 0.2$.

Supplementary Fig. 12: Colocalization between adult- eQTLs and pulse rate GWAS signals

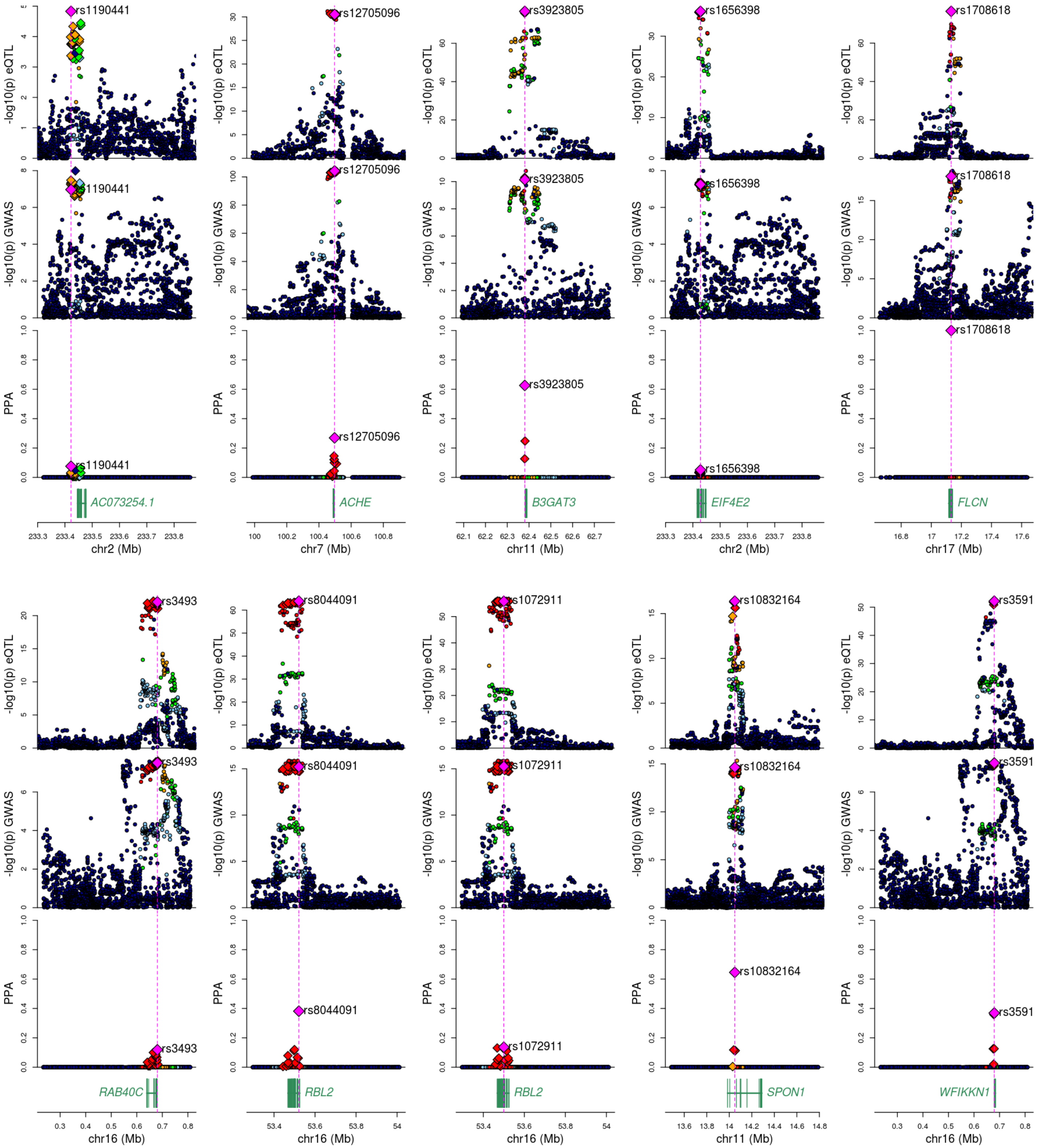

Plots showing -$\log_{10}$(p-value) adult- eQTL signals (top row), the colocalizing GWAS signals (pulse rate, middle row) and the PPA of each variant (bottom row) for seven eGenes (*ACHE*, *EIF4E2*, *FLCN*, *RAB40C*, *RBL2*, *SPON1* and *WFIKKN1*) and three eIsoforms (*AC073254.1*: ENST00000415506.6_2; *B3GAT3*: ENST00000531383.5_2; and *RBL2*:

ENST00000562850.1_1). The variant with the highest PPA of being causal for the colocalized eQTL and GWAS signals is shown as a magenta diamond. All other variants included in the 99% credible set are shown as red diamonds. The other eVariants are color-coded based on their LD (R^2, calculated using the 1000 Genomes) with the lead eVariant: 1) red: $R^2 \geq 0.8$; 2) orange: $0.6 \leq R^2 < 0.8$; 3) green: $0.4 \leq R^2 < 0.6$; 2) cyan: $0.2 \leq R^2 < 0.4$; 2) dark blue: $R^2 < 0.2$.

Supplementary Fig. 13: Comparison of the number of tissue-associated eQTLs and eGenes using two methods: single-tissue eQTLs versus mash

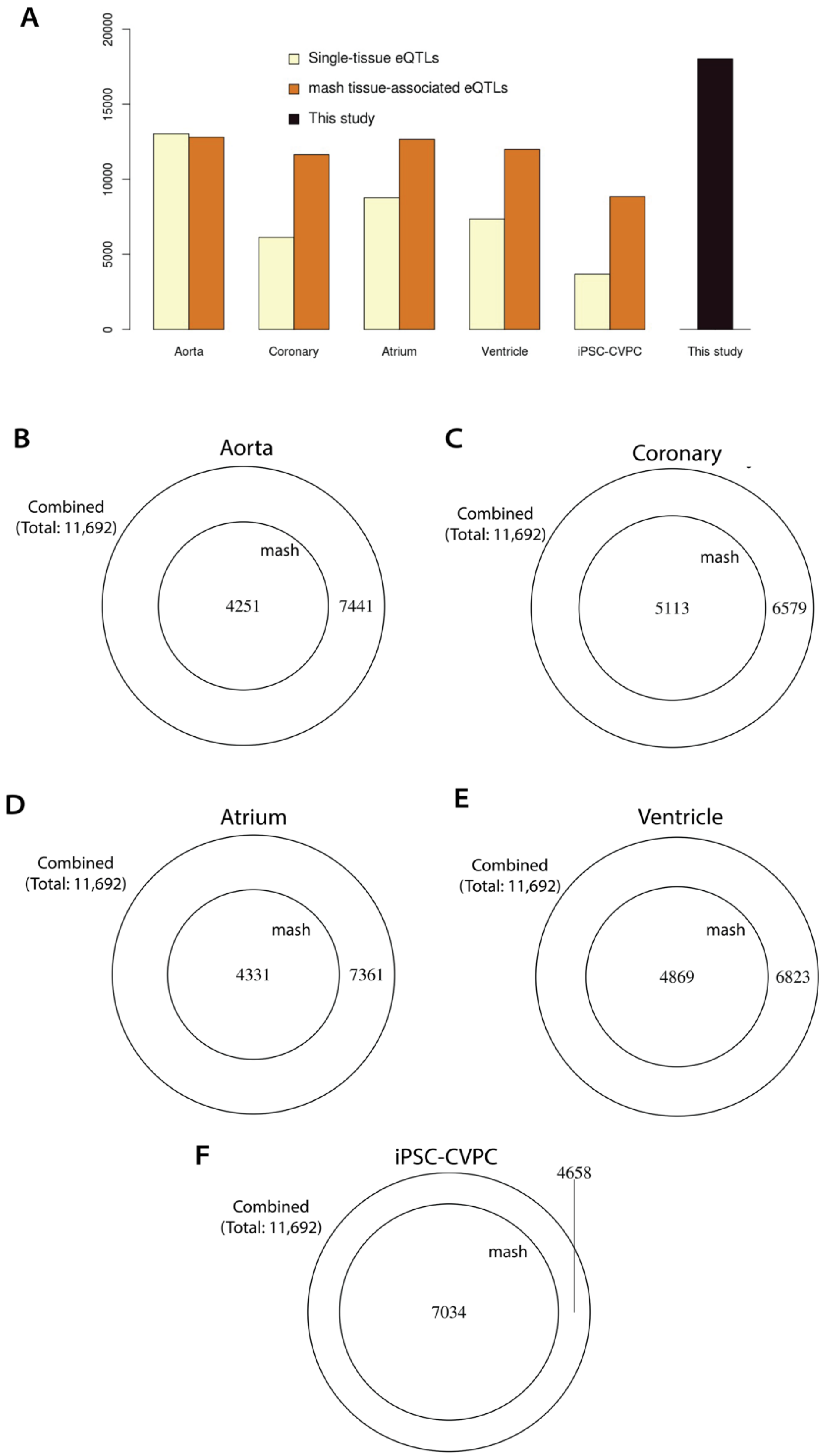

A) Barplots showing the number of eQTLs detected per tissue performing single-tissue eQTL analysis versus using mash on the eQTLs (for eGenes, ie, not for eIsoforms) identified in the first-step of the combined analysis (This study eQTLs) with their effect sizes and standard errors for each tissue from the single-tissue eQTL analyses as input.

B-F) Venn diagrams showing the overlap of the total 11,692 eGenes identified in the combined analysis with the eGenes identified as associated by mash (lfsr < 0.05) in each of the five tissues. To determine the overlap between these two sets, we intersected the 11,692 eGenes from the combined analysis with the eGenes identified as significant by mash (lfsr < 0.05) for the specific tissue. For example, for aorta (Panel B), mash identified 4,251 of the original 11,692 eGenes as being tissue-associated while the remaining 7,441 were not.

Supplementary Fig. 14: Enrichment of GWAS traits for tissue associations obtained using mash

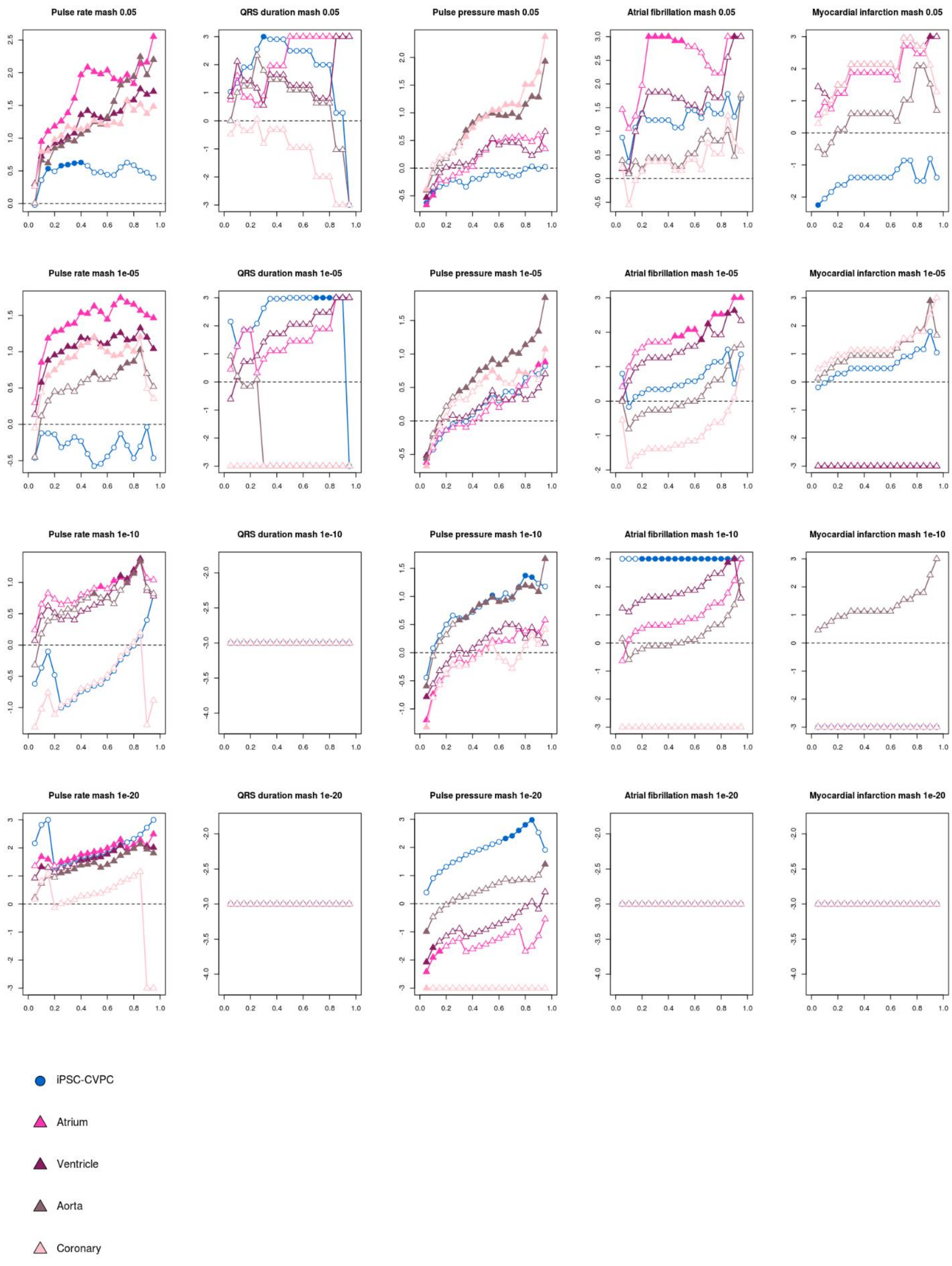

Enrichment for the associations between each of the five cardiac traits and diseases and tissue eQTL associations obtained using mash with multiple LFSR thresholds at multiple PP-H4 thresholds, as described in Donovan et al. [12]. Briefly, at each 0.05 PP-H4 increment between 0.05 and 0.95 (X axis), we tested the enrichment for each stage, organ or tissue for all the eGenes that had PP-H4 above the threshold using Fisher's exact test. The Y axis represents the $\log_2$ of the estimate, as calculated using the *fisher.test* function in R. Filled points represent p-values < 0.05. Log_2 estimate values > 3 or < -3 were set to +3 or -3, respectively.

In general, at local false sign rate (LFSR) < 0.05, MASH results in more associations with cardiac traits than our method, because it relies on thousands of eQTL signals, rather than a few hundred. At more stringent LFSR, enrichments are more consistent. For example, with our tissue-specific eQTL method, we found that pulse rate is associated with atrium, adult heart, adult eQTLs and, to a lower extent, arteria, whereas MASH at LFSR < 0.05 identified associations with all adult tissues. At the most stringent threshold (LFSR < 10^{-20}) MASH identified associations with atrium, ventricle and aorta.

Supplementary Fig. 15: Comparing fine mapping using colocalized eQTLs versus standard genetic fine mapping of GWAS loci

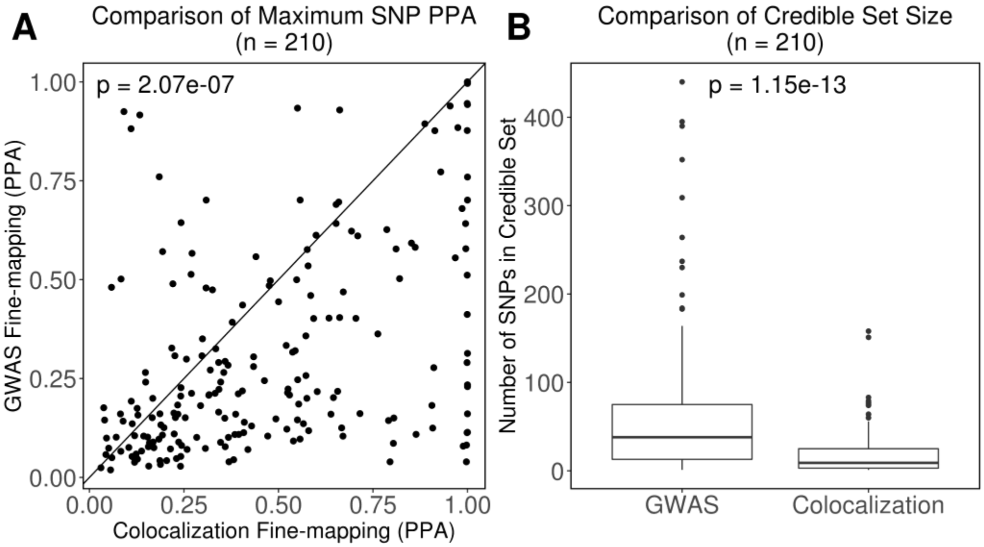

To determine if fine mapping using colocalized eQTLs provides an improvement in terms of credible set size and PPA of each lead variant compared with "standard genetic fine mapping" of GWAS loci, for each of the 210 loci we performed a genetic fine mapping using the GWAS signals alone. We observed that colocalizing GWAS signals with eQTLs reduces the credible set sizes ($p = 2.07 \times 10^{-7}$, paired t-test, two-sided) and results in stronger posterior probabilities for the lead variants ($p = 1.15 \times 10^{-13}$, paired t-test, two-sided), confirming that colocalization with eQTLs improves the identification of causal variants.

(A) Scatterplot showing the PPA of the lead variant obtained by fine mapping each of the 210 GWAS loci using cardiac eQTLs (X-axis, Supplementary Data 11) versus without using cardiac eQTLs (Y-axis, genetic fine mapping).

(B) Boxplots comparing the number of variants in each credible set between genetic fine mapping ("GWAS", left) and colocalization with cardiac eQTLs ("colocalization", right). The boxplots were built as follows: upper and lower edges represent the 25th and 75th percentiles and the middle line the median, vertical bars represent the distance from the 25th (or 75th) percentile minus (or plus) 1.5 times the interquartile range.

Supplementary Fig. 16: Associations between the overlap with chromatin states and PPA

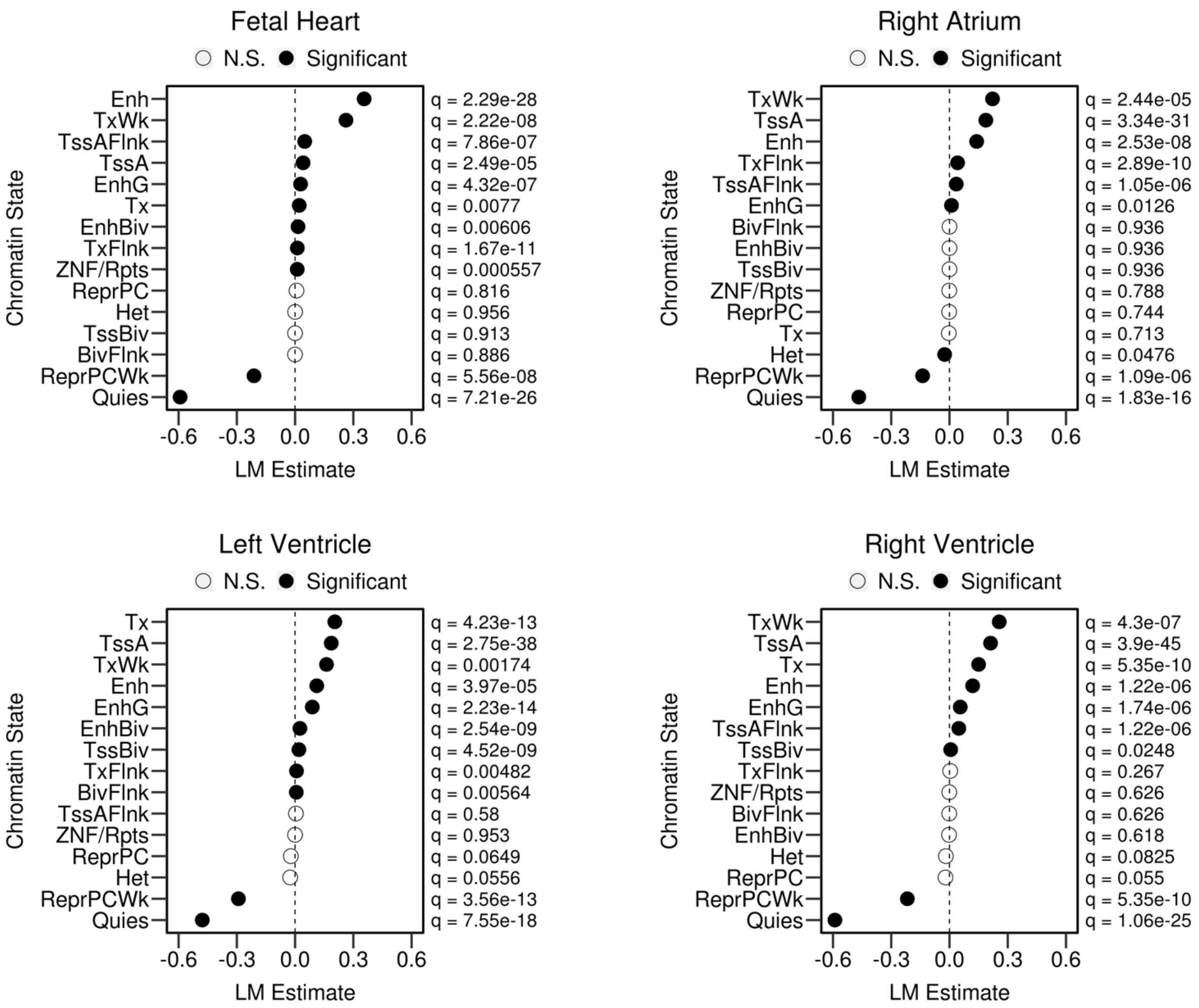

To examine the functional effects of the putative causal variants, we investigated the overlap of candidate causal variants with fetal and adult heart chromatin states. The plots show the linear regression p-values (*lm* function in R) between the overlap of variants in each of the 15 ChromHMM chromatin states and their PPA in the four cardiac tissues included in the Roadmap Epigenome. Filled circles represent FDR-corrected p-values (Benjamini-Hochberg). Variants with high PPA are more likely to reside in active chromatin states, such as enhancer (Enh) regions in fetal heart ($q = 2.29 \times 10^{-28}$) and transcriptional start sites (TssA) in adult heart (right atrium: $q = 3.34 \times 10^{-31}$; left ventricle: $q=2.75 \times 10^{-38}$; and right ventricle: $q=3.9 \times 10^{-45}$).

Supplementary Fig. 17: Association between each SNP's PPA and disease impact score

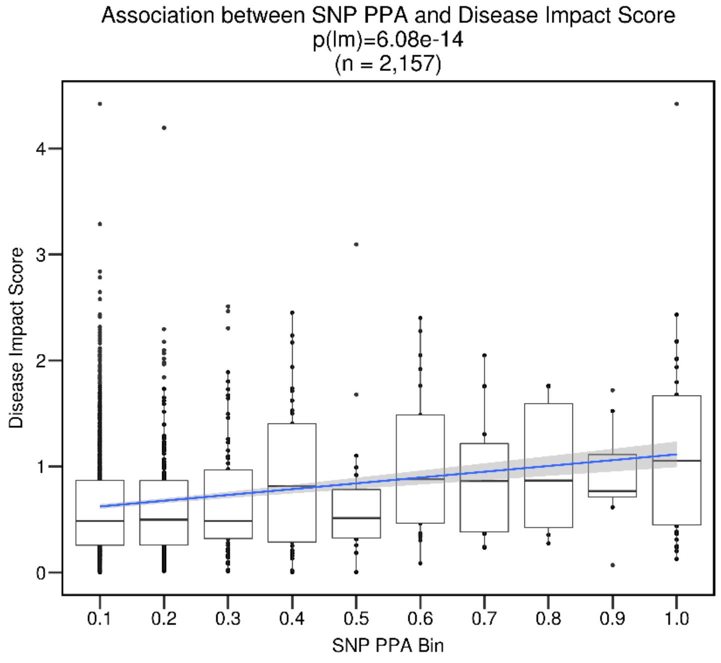

To examine the functional effects of the putative causal variants, we tested the association between the strength of the PPA for a putative causal SNP and the predicted impact of the SNP on disease. Boxplot showing the association between the PPA of each SNP and the disease impact score calculated using DeepSea [13,14]. The regression line ($p = 6.08 \times 10^{-14}$) was determined using linear regression (*lm* function in R). These results show that SNPs with higher PPAs are more likely to impact disease compared to SNPs with lower PPAs. The boxplots were built as follows: upper and lower edges represent the 25th and 75th percentiles and the middle line the median, vertical bars represent the distance from the 25th (or 75th) percentile minus (or plus) 1.5 times the interquartile range.

Supplementary Fig. 18: Assessing the optimal number of PEER factors

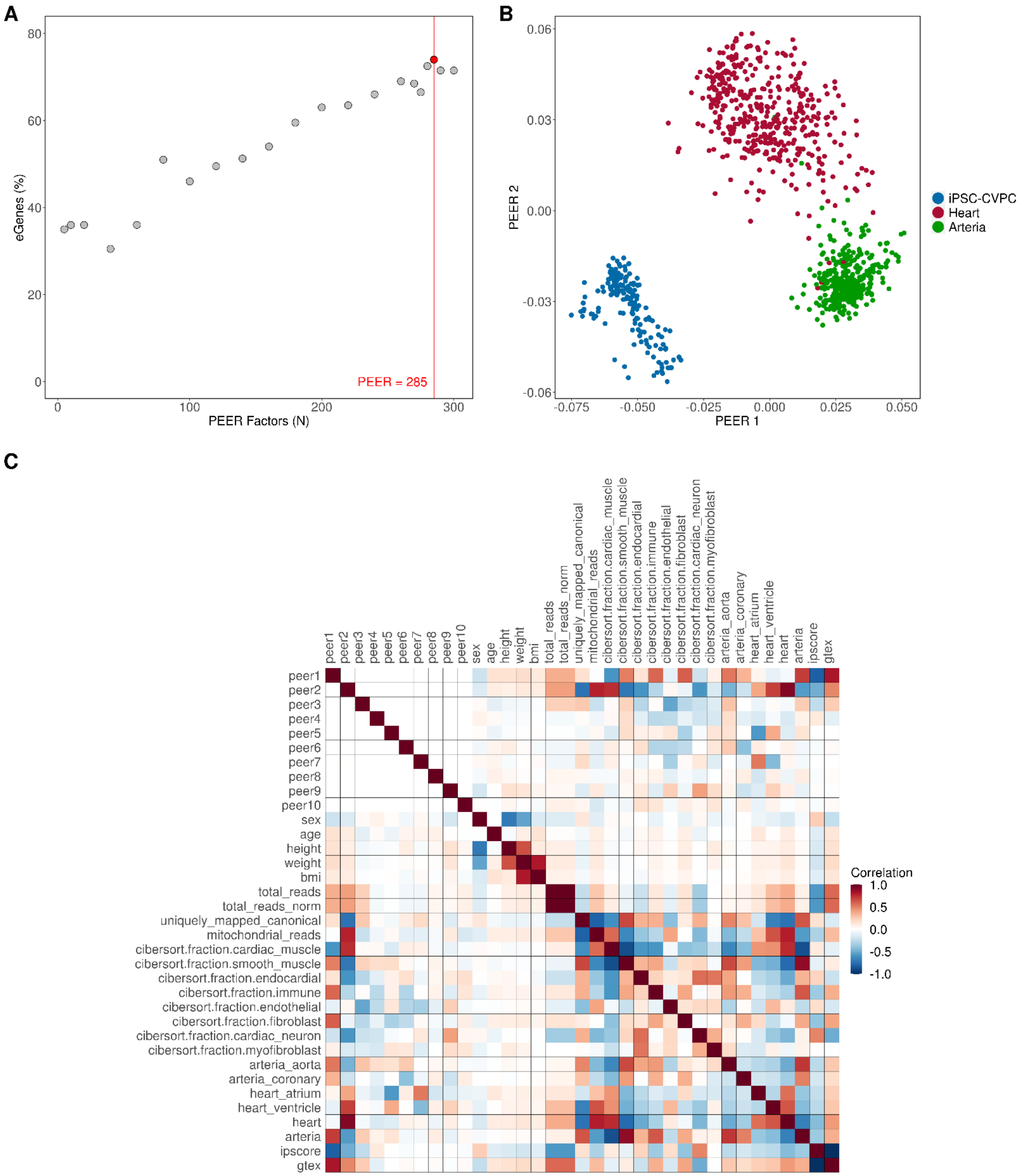

(A) Proportion of eGenes (Y axis) using different combinations of PEER factors (X axis: 5-300 PEER factors). The maximum number of eGenes is reached at 285 PEER factors. (B) Plot showing the PEER factor coordinates (PEER 1 and

PEER 2) of each of the 966 samples: the samples cluster by tissue, as expected. (C) Corrplot showing the correlation between all the covariates used to detect eQTLs, including PEER factors 1-10.

Consortium members

iPSCORE Consortium

University of California, San Diego, La Jolla, CA 92093, USA

Angelo D. Arias, Timothy D. Arthur, Paola Benaglio, Victor Borja, Megan Cook, Matteo D'Antonio, Agnieszka D'Antonio-Chronowska, Christopher DeBoever, Margaret K.R. Donovan, KathyJean Farnam, Kelly A. Frazer, Kyohei Fujita, Melvin Garcia, Olivier Harismendy, David Jakubosky, Kristen Jepsen, Isaac Joshua, He Li, Hiroko Matsui, Naoki Nariai, Jennifer P. Nguyen, Daniel T. O'Connor, Jonathan Okubo, Fengwen Rao, Joaquin Reyna, Lana Ribeiro Aguiar, Bianca Salgado, Nayara Silva, Erin N. Smith, Josh Sohmer, Shawn Yost, William W. Young Greenwald

Salk Institute for Biological Studies, La Jolla, CA 92037, USA

Athanasia D. Panopoulos, W. Travis Berggren, Kenneth E. Diffenderfer

Supplementary References

1. Engstrom, P.G. *et al.* Complex Loci in human and mouse genomes. *PLoS Genet* **2**, e47 (2006).
2. Yelin, R. *et al.* Widespread occurrence of antisense transcription in the human genome. *Nat Biotechnol* **21**, 379-86 (2003).
3. Chung, J. *et al.* Genome-wide pleiotropy analysis of neuropathological traits related to Alzheimer's disease. *Alzheimers Res Ther* **10**, 22 (2018).
4. Skuodas, S. *et al.* The ABCF gene family facilitates disaggregation during animal development. *Mol Biol Cell* **31**, 1324-1345 (2020).
5. Lu, A. *et al.* Fast and powerful statistical method for context-specific QTL mapping in multi-context genomic studies. *bioRxiv*, 2021.06.17.448889 (2021).
6. Consortium, G.T. The GTEx Consortium atlas of genetic regulatory effects across human tissues. *Science* **369**, 1318-1330 (2020).
7. Stephens, M. False discovery rates: a new deal. *Biostatistics* **18**, 275-294 (2017).
8. Urbut, S.M., Wang, G., Carbonetto, P. & Stephens, M. Flexible statistical methods for estimating and testing effects in genomic studies with multiple conditions. *Nat Genet* **51**, 187-195 (2019).
9. He, Y. *et al.* sn-spMF: matrix factorization informs tissue-specific genetic regulation of gene expression. *Genome Biol* **21**, 235 (2020).
10. Hocker, J.D. *et al.* Cardiac cell type-specific gene regulatory programs and disease risk association. *Sci Adv* **7**(2021).
11. Kim-Hellmuth, S. *et al.* Cell type-specific genetic regulation of gene expression across human tissues. *Science* **369**(2020).
12. Donovan, M.K.R., D'Antonio-Chronowska, A., D'Antonio, M. & Frazer, K.A. Cellular deconvolution of GTEx tissues powers discovery of disease and cell-type associated regulatory variants. *Nat Commun* **11**, 955 (2020).
13. Zhou, J. & Troyanskaya, O.G. Predicting effects of noncoding variants with deep learning-based sequence model. *Nat Methods* **12**, 931-4 (2015).
14. Zhou, J. *et al.* Deep learning sequence-based ab initio prediction of variant effects on expression and disease risk. *Nat Genet* **50**, 1171-1179 (2018).
15. D'Antonio, M. Fine mapping spatiotemporal mechanisms of genetic variants underlying cardiac traits and disease. *figshare*, https://doi.org/10.6084/m9.figshare.c.5594121 (2021).
16. Buniello, A. *et al.* The NHGRI-EBI GWAS Catalog of published genome-wide association studies, targeted arrays and summary statistics 2019. *Nucleic Acids Res* **47**, D1005-D1012 (2019).
