# Supplementary material for: Fine mapping spatiotemporal mechanisms of genetic variants underlying cardiac traits and disease
Source: Nat Commun. 2023 Feb 28;14:1132. doi: 10.1038/s41467-023-36638-2 (PMC9975214; doi:10.1038/s41467-023-36638-2)
Supplement: Supplementary file 3 — Description of Additional Supplementary Files [file 41467_2023_36638_MOESM3_ESM.pdf]

Description of Additional Supplementary Files

File Name: Supplementary Data 1
Description: **Sample metadata**
The table shows sample information for all 966 RNA-seq samples which were used for eQTL analysis, including: sample ID (UUID for iPSCORE samples, SRA run ID for GTEx samples), whole genome sequencing ID (UUID for iPSCORE samples, subject ID for GTEx samples); subject ID; subject name; source study (iPSCORE or GTEx); organ (arteria, heart or iPSC-CVPC); tissue (aorta, coronary artery, atrial appendage, left ventricle or iPSC-CVPC); normalized read depth (calculated as the number of reads in each sample divided by the mean number of reads across all samples); % of mitochondrial reads; unique differentiation identifier (UDID, used only for iPSC-CVPC); sex; and cell types deconvoluted using CIBERSORT. Additional covariates used for the eQTL analysis, including 20 genotype PCs and PEER factors, are reported in Figshare (https://doi.org/10.6084/m9.figshare.c.5594121).

File Name: Supplementary Data 2
Description: **eGenes and eIsoforms**
The table describes the lead variant for each expressed gene and isoform. For each gene or isoform, shown are: phenotype (gene or isoform), transcript ID (from Gencode V.34lift37; for genes, transcript ID is the same as gene ID), gene ID, gene name, gene type (as defined by Gencode); whether the tested eVariant is primary or conditional, the SNP ID, its chromosome, position, reference allele, alternative allele, SNP ID; beta, standard error of beta and p-value, calculated using limix; number of tests (i.e. number of independent variants tested for the selected gene, calculated using eigenMT) and FDR-corrected p-value calculated by eigenMT; q-value (Benjamini-Hochberg-corrected p-values); and whether the tested gene or isoform is an eGene ("Yes" for all genes and isoforms with q-value ≤ 0.05). Full summary statistics for all genes and isoforms are reported in Figshare (https://doi.org/10.6084/m9.figshare.c.5594121).

File Name: Supplementary Data 3
Description: **Colocalization between each eIsoform and its associated eGene**
For each of the 5,744 eIsoforms whose associated gene is an eGene, the Table shows all the colocalization between isoform eQTL and gene eQTL signals as calculated using the *coloc.abf* in the R package *coloc*: transcript ID, gene ID, the type of eQTL for the eIsoform and the eGene, the posterior probability of each hypothesis, the variant with the highest posterior probability of being causal and its posterior probability. The five hypotheses are: 1) H0: neither eIsoform nor eGene has a significant association at the tested locus; 2) H1: only the eIsoform is associated; 3) H2: only the eGene is associated; 4) H3: both eIsoform and eGene eQTL signals are associated but the underlying variants are different; and 5) H4: both eIsoform and eGene eQTL signals are associated and share the same underlying variants. Full PPAs between each pair of eGenes or eIsoforms are reported in Figshare (https://doi.org/10.6084/m9.figshare.c.5594121).

File Name: Supplementary Data 4
Description: **Interactions between eQTL signals and stage, organ, tissue or cell type**
The Table shows all the interactions between eQTLs and stage (iPSC-CVPC or adult), organ (arteria or heart), tissue (atrium, ventricle, aorta or coronary artery) or cell type (cardiac muscle, smooth muscle, endocardial, immune, endothelial, fibroblast, cardiac neuron or myofibroblast).

For each eQTL, shown are: phenotype (gene or isoform), transcript ID (from Gencode V.34lift37; for genes, transcript ID is the same as gene ID), gene ID, whether the eVariant is primary or conditional, the SNP ID, the interaction; effect size, standard error, p-value and Bonferroni-adjusted p-value for the interaction between genotype and stage, organ, tissue or cell type; effect size, standard error, p-value and Benjamini-Hochberg-adjusted p-value for the tested stage, organ or tissue, or the top quartile for cell types; effect size, standard error, p-value and Benjamini-Hochberg-adjusted p-value for all the other stages, organs or tissues, or the bottom quartile for cell types; whether the eQTL is associated with stage, organ, tissue or cell type, whether it is specific or associated (as shown in Supplementary Fig. 1, Supplementary Fig. 2 and Supplementary Fig. 3). Only significant interactions are shown. The full table is reported in Figshare (https://doi.org/10.6084/m9.figshare.c.5594121).

File Name: Supplementary Data 5
Description: **Enrichment of cell type- eQTLs for cell type-associated snATAC peaks**
The table shows the enrichments of eQTLs associated with each cell type and cell type-associated snATAC-seq peaks from an independent study 10. For each cell type in the eQTL analysis, we performed a paired t-test between the relative accessibility score (RAS) value for each cell type in the snATAC-seq dataset and the mean value across all other cell types for the cell type-associated eGenes. The Table shows: phenotype (gene or isoform); cell type in the eQTL analysis and in the snATAC-seq analysis; estimate, 95% confidence interval and p-value calculated using the *t.test* (paired, two-sided) function in R.

File Name: Supplementary Data 6
Description: **eVariants shared between multiple eGenes or eIsoforms**
The Table shows the eVariants that are shared between multiple eGenes or eIsoforms. For each eVariant, we indicate the number of associated genes (eGenes or genes with associated eIsoforms) and their gene name. Full PPAs between each pair of eGenes or eIsoforms are reported in Figshare (https://doi.org/10.6084/m9.figshare.c.5594121).

File Name: Supplementary Data 7
Description: **Colocalization between eQTLs and GWAS signals**
For each eGene and eIsoform overlapping genome-wide significant GWAS loci, the Table shows colocalization PPAs. Specifically, shown are: trait ID, trait name; transcript ID, gene ID, gene name; whether the eVariant is primary or conditional; phenotype (“gene” or “isoform”); the SNP ID (chromosome, position, reference and alternative allele, separated by underscores) and PPA for the SNP with the strongest PPA; and the PPA for each of the five colocalization hypotheses.

File Name: Supplementary Data 8
Description: **GWAS traits enrichment for stage, organ, tissue and cell type- eQTLs**
The Table shows the enrichment analysis for the colocalization between stage, organ, tissue and cell type- eQTLs and each of the five GWAS traits: trait ID, trait name (as in Supplementary Data 7); tested interaction, as described in Supplementary Data 4; estimate, confidence interval, log2 ratio of the estimate and p-value, as calculated using the *fisher.test* (two-sided) function in R. the data described in this table was used as input to create Figure 5E-G, Supplementary Fig. 9 and Supplementary Fig. 10.

File Name: Supplementary Data 9
Description: **Single-tissue eQTLs**
The Table shows the number of expressed genes, eGenes with primary and conditional eQTLs, the total number of eQTL signals and the total number of conditional eQTL signals for each of the five tissues (atrium, ventricle, aorta, coronary artery and iPSC-CVPCs). The full summary statistics for each tissue are reported in Figshare (https://doi.org/10.6084/m9.figshare.c.5594121).

File Name: Supplementary Data 10
Description: **Tissue associations detected by mash**
The Table shows, for multiple lfsr thresholds, the number of eQTL signals associated with each tissue. The full mash output, including lfsr values for each eQTL, is reported in Figshare (https://doi.org/10.6084/m9.figshare.c.5594121).

File Name: Supplementary Data 11
Description: **Fine mapped cardiac GWAS loci**
The Table shows each of the 331 colocalizations between eQTL and GWAS signals. Since multiple eQTL signals may map to the same GWAS signal, we calculated LD between each pair of lead SNPs and obtained 210 clusters of eQTL signals (all SNPs in each cluster had D' > 0.8). For each of these clusters, we selected the eQTL signal associated with the smallest 99% credible set and used it to fine map its corresponding GWAS signal. This table reports all these colocalizations and the information about 99% credible sets associated with each colocalization: trait ID, trait name; phenotype ("gene" or "isoform"); transcript ID, gene ID, gene name; whether the tested variant is primary or conditional; the PPA for each of the five colocalization hypotheses; the SNP ID, RS ID and PPA for the SNP with the strongest PPA; the number of variants in the 99% credible set; the LD cluster ID (chromosome and cluster, separated by underscore); whether the colocalization was used for fine mapping; RS ID of the SNP in highest LD in the GWAS catalog (frozen at June 2, 2021), its R2 and D' values, calculated using LDlink (https://ldlink.nci.nih.gov/), the Pubmed IDs associated with the SNP in the GWAS catalog and the category of association with the GWAS catalog. These categories are (Figure 6F): 1) "in catalog", if the SNP with the highest PPA has previously been found in other GWAS for the same trait; 2) "high R2", if the SNP with the highest PPA is in high LD (R2 ≥ 0.8) with a SNP associated with the same trait in the GWAS catalog; 3) "high D'", if the SNP with the highest PPA is in high LD (D' ≥ 0.8 and R2 < 0.8) with a SNP associated with the same trait in the GWAS catalog; and 4) empty, if the SNP with the highest PPA is not in high LD with any SNP associated with the same trait in the GWAS catalog. Full information about each colocalization, including the summary statistics of the GWAS at each locus, the associated eQTL summary statistics, the PPA of each tested SNP and the composition of each credible set, has been deposited to Figshare (https://doi.org/10.6084/m9.figshare.c.5594121).
